# Interest and Limits of [^18^F]ML-10 PET Imaging for Early Detection of Response to Conventional Chemotherapy

**DOI:** 10.3389/fonc.2021.789769

**Published:** 2021-12-20

**Authors:** Elodie Jouberton, Sébastien Schmitt, Aurélie Maisonial-Besset, Emmanuel Chautard, Frédérique Penault-Llorca, Florent Cachin

**Affiliations:** ^1^ Service de Médecine Nucléaire, Centre Jean PERRIN, Clermont-Ferrand, France; ^2^ Imagerie Moléculaire et Stratégies Théranostiques, UMR1240, Université Clermont Auvergne, INSERM, Clermont-Ferrand, France; ^3^ Service de Pathologie, Centre Jean PERRIN, Clermont-Ferrand, France

**Keywords:** [^18^F]ML-10, positron emission tomography, early evaluation, apoptosis, oncology

## Abstract

One of the current challenges in oncology is to develop imaging tools to early detect the response to conventional chemotherapy and adjust treatment strategies when necessary. Several studies evaluating PET imaging with 2-deoxy-2-[^18^F]fluoro-D-glucose ([^18^F]FDG) as a predictive tool of therapeutic response highlighted its insufficient specificity and sensitivity. The [^18^F]FDG uptake reflects only tumor metabolic activity and not treatment-induced cell death, which seems to be relevant for therapeutic evaluation. Therefore, to evaluate this parameter *in vivo*, several cell death radiotracers have been developed in the last years. However, few of them have reached the clinical trials. This systematic review focuses on the use of [^18^F]ML-10 (2-(5-[^18^F]fluoropentyl)-2-methylmalonic acid) as radiotracer of apoptosis and especially as a measure of tumor response to treatment. A comprehensive literature review concerning the preclinical and clinical investigations conducted with [^18^F]ML-10 was performed. The abilities and applications of this radiotracer as well as its clinical relevance and limitations were discussed. Most studies highlighted a good ability of the radiotracer to target apoptotic cells. However, the increase in apoptosis during treatment did not correlate with the radiotracer tumoral uptake, even using more advanced image analysis (voxel-based analysis). [^18^F]ML-10 PET imaging does not meet current clinical expectations for early detection of the therapeutic response to conventional chemotherapy. This review has pointed out the challenges of applying various apoptosis imaging strategies in clinical trials, the current methodologies available for image analysis and the future of molecular imaging to assess this therapeutic response.

## Introduction

One of the current challenges in the management of cancer patients is the early evaluation of response to therapies in order to adjust treatment strategies when necessary. To date, there is no consensus on the nature of the best method to be used in this setting.

Several approaches are currently explored, particularly in the field of functional imaging with the development of non-invasive tools for the prediction and evaluation of therapeutic response. Limited sensitivity and specificity of clinical evaluation has been highlighted and sequential tissue biopsies are not ethically feasible (1). Furthermore, no circulating biomarkers such as cell free DNA are currently clinically validated. In this context, functional and molecular imaging modalities, such as Positron Emission Tomography (PET) or Single-Photon Emission Computed Tomography (SPECT), are under investigation. These imaging modalities can assess vascular, metabolic, biochemical and molecular events in cancer cells, which occur upstream of morphological changes, allowing an early assessment of the therapeutic response. In oncology, the most commonly used radiopharmaceutical is the 2-[^18^F]-fluoro-2-deoxy-D-glucose ([^18^F]FDG), whose uptake is regulated by glucose metabolism (2). The value of PET imaging with [^18^F]FDG during the diagnostic phase but also for the follow-up of treatments in many cancers has already been demonstrated. Several studies have explored biological parameters correlated with tumor response. For example, Groheux et al. demonstrated, in 78 Triple Negative Breast Cancer (TNBC) patients, that reduction of the [^18^F]FDG uptake (ΔSUVmax), assessed by PET/CT, performed after only 2 cycles of neoadjuvant therapy (NAT), reliably predicts pathological complete response (pCR). However, the optimal ΔSUVmax for prediction of pCR was therapy-specific, being almost 2-fold higher in a dose-dense regimen than in the classical anthracycline-taxane sequence (-68% vs. -35%, respectively) (3). These trials have shown promising results confirming the interest of this radiotracer as a tool for the early selection of responder patients.

However, the number of false positive (FP) and false negative (FN) cases is still too high to redirect the therapeutic strategy only based on [^18^F]FDG PET imaging findings. These artefacts can be due to the incapacity of [^18^F]FDG to detect cancer cells in low number or with low glucose metabolism (FN), turn-off of glycolysis without subsequent cell death (FN) or presence of intense inflammatory infiltrate with high metabolism (FP) (4, 5). In addition, one limitation is the inability of [^18^F]FDG to discriminate dead cells from metabolically inactive cells in response to chemotherapy.

Consequently, several other radiotracers have been investigated to complement the biological information provided by the [^18^F]FDG and thus improve the performance of functional imaging for the early evaluation of therapeutic response. For example, [^18^F]fluoro-3′-deoxy-3′-L-fluorothymidine ([^18^F]FLT) was evaluated for the visualization of cell proliferation or [^99m^Tc]Annexin V for the measurement of apoptosis (6, 7). Apoptosis or programmed cell death is a physiological process by which supernumerary or dysfunctional cells are eliminated from the body. Apoptosis plays a key role in several biological phenomena such as embryonic development, cell homeostasis, ageing, the body’s defense against tumor cells and viral and bacterial infections. Apoptosis is an “orderly” cell death, resulting from a highly regulated programme that induces the destruction of the cell while preserving the surrounding cellular integrity, without inducing any mesenchymal reaction typically seen to fibrosis. It is also described as a silent death which, unlike necrosis, does not cause an inflammatory reaction. It is a very rapid phenomenon (within 48 h following treatment initiation) defined by a highly characteristic set of cellular events, including among others, an initiation stage, which is reversible at the beginning (8). Various stimuli can initiate apoptosis, notably the action of cytotoxic drugs. This stage leads to the activation of a family of proteases called caspases. Finally, the execution phase occurs, involving the mitochondria, proteins of the Bcl-2 family, and caspases. The last stage is irreversible, and allows the degradation of intracellular constituents, by fragmentation and formation of apoptotic bodies. By preserving the integrity of the plasma membrane, this process generates early no inflammation, and the apoptotic cell is rapidly phagocyted thanks to the externalisation of phosphatidylserins (PS) residues. During this last step, the apoptotic cell undergoes numerous morphological changes.

Apoptosis can mainly be triggered by two different pathways: *via* death receptors (extrinsic pathway) expressed at the surface of cells or by the mitochondria through internal signals (intrinsic pathway) (9). The extrinsic apoptotic pathway is triggered by the activation of cell death receptors such as tumor necrosis factor (TNF) which, through its receptor (TNFR), stimulates the initiating procaspases to form the death-inducing signalling complex (DISC). This triggers the proteolytic cascade, which will simulate effector caspases to produce lysis of cellular proteins. The intrinsic pathway is activated by cellular stressors, which cause the release of cytochrome c and other proteins from mitochondria into the cytosol. Cytochrome c induces the formation of the so-called apoptosome complex which activates caspase 9 and the protease hydrolysis cascade. Apoptosis is regulated by the Bcl family of proteins. These include pro-apoptotic agents, such as Bax and Bak, and anti-apoptotic agents, such as Bcl proteins. Various stimuli can initiate apoptosis, notably the action of cytotoxic drugs. Indeed, is currently recognized as the major form of cell death induced by chemotherapies, such as etoposide, dexamethasone, paclitaxel, 5’-fluoro-deoxyuridine, 5’-fluorouracil, and adriamycin (10–14). The literature review shows that the induction of apoptosis by conventional chemotherapy treatments is triggered by the expression of death receptor ligands, particularly the Fas ligand (FasL), but also by inducing the release of cytochrome c from the mitochondria (15). Their apoptotic effect has been demonstrated in several cancer cell lines *in vitro*, but also *in vivo*, in mouse models of mammary adenocarcinoma following treatment with cyclophosphamides (16). Currently, histological examinations are not suitable to detect early apoptosis during therapy protocols. However, this parameter may be monitored by nuclear imaging, a non-invasive and sensitive method allowing assessment of either of the tumor or the putative metastatic sites. Many radiotracers have been developed for *in vivo* imaging of apoptosis such as PS exposure using Annexin V radiolabeled with technetium-99m, fluorine-18 or gallium-68, activation of caspases with isatins radiolabelled with fluorine-18 or mitochondrial outer membrane permeabilization using [^18^F]fluorobenzyl triphenylphosphonium cation (17–21) (**Table 1**). Indeed, one of the most extensively studied approaches for imaging apoptosis relies on the high affinity of annexin V for PS present on the membrane surface of apoptotic cells. The [^99m^Tc]hydrazinonicotinamide-annexin V ([^99m^Tc]HYNIC-annexin V) has been used for assessment of therapeutic responses in many solid tumors and hematologic cancers (62). The results of these clinical trials revealed a high tracer affinity for the PS, and a correlation between tracer accumulation in cancer cells and number of dying cells. Nevertheless due to its poor ability to predict treatment response in various tumor types and the difficulty to distinguish apoptotic cells from necrotic cells, the clinical use of [^99m^Tc]HYNIC-annexin V remained very limited (22). The second major radiotracer family that has been widely studied for imaging apoptosis *in vivo* are inhibitors or substrates of caspases 3. Caspases represent attractive targets because of their central role in the apoptotic pathway including the death receptor pathway and the mitochondrial pathway that converge on effector caspases, particularly the executive enzymes caspase-3/7 that recognise and cleave the DEVD peptide sequence. Although the radiotracers targeting caspases are promising, Spires-et al. found that caspase 3 activation was not necessarily restricted to apoptosis (31). These authors showed that caspase 3 was required for the full activation of several important physiological cellular functions, including platelet aggregation and enzyme secretion from pancreatic acinar cells. The 2-(5-fluoropentyl)-2-methylmalonic acid radiolabelled with fluorine-18 ([^18^F]ML-10) is part of a new family of low molecular weight derivatives developed by ApoSense. This radiotracer was first validated in several preclinical models and has been further tested in several clinical trials.

**Table 1:** Example of radiotracers under evaluation for the scintigraphic imaging of apoptosis.

Molecular type	Radionuclide	Target	Development phase	Advantages (+) or d disadvantages (-)	Reference
Annexin V	^18^F ^68^Ga ^99m^Tc	PS	Clinical trials (III)	+: Allows assessment of early therapeutic response. -: Non-specific binding in necrotic cells. High digestive clearance preventing visualisation of abdominal and pelvic metastases.	(18, 22–30)
ICMT-11 CP18 MICA-302	^18^F	Inhibitors/substrates of caspase 3	Clinical trials (I)	+: Absence of toxicity. Favourable dosimetry. Rapid biodistribution. -: High renal, hepatic, intestinal and urinary clearance. Caspase 3 not specific for apoptosis.	(31–40)
Synaptotagmin	^99m^Tc ^111^In	PS	Preclinical studies	+: Higher affinity compared to annexin V. -: Sames disadvantages as Annexin V	(41, 42)
Annexin B1	^18^F ^99m^Tc	PS	Preclinical studies	-: Induces an immune response. Affinity comparable to annexin V.	(43, 44)
Zn-DPA probe	^99m^Tc ^18^F	PS	Preclinical studies	-: Affinity comparable to annexin V.	(45)
Lactadherin	^99m^Tc	PS	Preclinical studies	+: Higher affinity compared to annexin V. -: Important uptake of radioactivity in the liver and intestine.	(46)
Bavituximab	^124^I	beta-2 glycoprotein-1 (β2-GP1)	Clinical trials (I)	+: Absence of toxicity. -: Low tumor uptake.	(47)
Duramycin	^99m^Tc	PE	Preclinical studies	-: Cellular uptake mechanism not elucidated yet.	(48–50)
FBnTP sestaMIBI	^99m^Tc	Transmembrane potential	Preclinical studies	-: Negative contrast tracer of dying cells, Complicated analysis of results. Cellular uptake not always correlated with apoptotic fraction.	(51, 52)
PARPi Olaparib	^18^F	PARP	Preclinical studies	+: High gastrointestinal clearance. Binding in a few immune cells.	(53–56)
Anti-γH2AX-TAT	^89^Zr	γH2AX	Preclinical studies	+: Allows assessment of early therapeutic response. -: Induces an immune response. Low signal-to-noise contrast.	(57–59)
Apopep-1 CQRPPR	^124^I	Histone	Preclinical studies	-: Low *in vivo* metabolic stability.	(60, 61)

In this review, an overview of the current state of preclinical and clinical [^18^F]ML-10 development is provided and its value and limitations as an apoptosis radiotracer are discussed.

## [^18^F]ML-10 As an Apoptosis Radiotracer

[^18^F]ML-10 is an organic molecule, with a low molecular weight (205 daltons), composed of a malonic acid scaffold bearing a methyl group and a fluoropentyl chain. As the malonic group allows the recognition with apoptotic cells, fluorine-18 is advantageously incorporated on the aliphatic side chain.

[^18^F]ML-10 shows selective accumulation within apoptotic cells compared to healthy or necrotic cells in response to a set of cellular changes, characteristic of apoptotic-specific membrane alterations that occur early in the apoptosis process, at stages where membrane integrity is still preserved. The selectivity of [^18^F]ML-10 for apoptotic cells can be partly attributed to the detection of permanent and irreversible molecular changes during the apoptotic phenomena (63).

- Exposure of phosphatidylserins to the cell surface leading to acidification of the extracellular medium in contact with the outer membrane. This change in pH enables the capture of protons by the malonate group resulting in increased hydrophobicity and charge dispersion, facilitating the penetration of the radiotracer into the membrane. Indeed, at physiological pH, the di-anionic form of the carboxylic acid functions predominates (pKa: 5.78 and 3.07) and explains its high solubility in aqueous media. After mono-protonation, favored by the phenomenon of apoptosis, the radiotracer becomes a mono carboxylate derivative. Consequently, the remaining negative charge can be distributed over the four oxygen atoms and this dispersion of charge promotes interaction of the molecule with the membrane interface (63).

- Alteration of the mitochondrial transmembrane potential leading to the irreversible depolarization of the membrane. Hence, the diffusion of the radiotracer towards the inner membrane of the apoptotic cell is facilitated by the loss of the plasma membrane asymmetry.

- The irreversible loss of control of the intracellular pH, leading to acidification of the cytosol and sequestration of the radiotracer in the apoptotic cell, due to the binding *via* electrostatic and hydrophobic interactions to cytoplasmic proteins.

Cohen et al. have shown that the behavior of [^3^H]ML-10 is modified during alterations of membrane potential or pH of healthy cells (63). Depolarization followed by repolarization of a viable cell results in the uptake and subsequent exclusion of [^3^H]ML-10 from the cell. On one hand, the radiotracer uptake by the viable cell, subsequent to the acidification of the medium, is only transient. On the other hand, the phenomenon of apoptosis is associated with the permanent loss of these vital regulatory functions and lead to the uptake of [^18^F]ML-10 (64). To date, the mechanism of uptake of the [^18^F]ML-10 remains challenging and warrants further investigations. Therefore, the interpretation of some of the *in vitro* and *in vivo* results obtained in the various preclinical and clinical studies is difficult and conclusions remain speculative. Nevertheless, several advantages of [^18^F]ML-10 have led to its transfer and use in clinical research. Among them, we can highlight: i) its favourable biodistribution profile with no radioactive signal in viable cells, ii) its rapid urinary clearance and iii) its specificity for apoptotic cells.

## Automated [^18^F]ML-10 Radiolabeling

**Figure 1:**
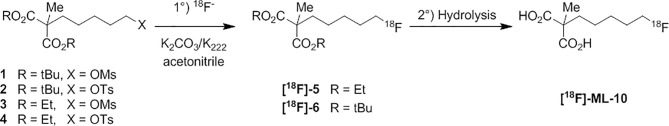
Automated radiosyntheses of [^18^F]ML-10.

[^18^F]ML-10 radiosynthesis was typically performed according to a two-step procedure: radiofluorination by nucleophilic substitution of a sulfonate precursor using the classical ^18^F^-^/K_2_CO_3_/K_222_ complex followed by hydrolysis of the protective groups to obtain [^18^F]ML-10 after purification and formulation steps (**Figure 1**).

The first automated radiosynthesis of [^18^F]ML-10 was described in 2008 by Reshef et al. starting from a mesylate precursor **1** protected by *tert*-butyl esters (65) (**Table 2**). After radiofluorination at 90°C in acetonitrile for 15 min, esters were cleaved by hydrolysis under acidic conditions (TFA/H_2_O) at room temperature for 15 min. [^18^F]ML-10 was then obtained in 30-40% decay-corrected radiochemical yield (d.c. RCY) with a radiochemical purity (RCP) greater than 99% and a molar activity (A_m_) higher than 40.7 GBq/µmol, after semi-preparative RP-HPLC purification and SPE (Sep-pak C18 cartridge) formulation.

**Table 2:** Automated radiosyntheses of [^18^F]ML-10.

Precursor	Automate	Conditions	Purification method (conditions)	Radiotracer	Reference
**1**	nd	1°) 90°C 15 min 2°) TFA/H_2_O rt, 15 min	Semi preparative RP-HPLC^a^ (H_2_O/CH_3_CN/TFA 80:20:1 10 mL/min) and SPE	RCY : 30-40% RCP > 99% A_m_: 40.7 GBq/µmol t = 75 min	(65)
	TRACERlab FX-FN	1°) 90°C 15 min 2°) 3M HCl, 115°C,15 min	Semi preparative RP-HPLC^b^ (H_2_O/CH_3_CN/AcOH 70:30:0.5 16 mL/min)	RCY: 39.8 ± 8.7% RCP > 99% A_m_: 235 ± 85 GBq/µmol t = 70-75 min	(66)
	TRACERlab FX-FN	1°) 120°C 20 min 2°) 2 M HCl, 120°C, 20 min	Semi preparative RP-HPLC^c^ (H_2_O/CH_3_CN/TFA 65:35:0.01 5 mL/min)	RCY: 26-31% RCP > 99% A_m_: 25.3 GBq/µmol t = 80 min	(67)
	SynChrom R&D EVOI	1°) 90°C 15 min 2°) 3M HCl, 115°C, 15 min	Semi preparative RP-HPLC^d^ (H_2_O/CH_3_CN/AcOH 70:30:0.5 3 mL/min)	RCY : 23.3 ± 10.8% RCP > 99% A_m_ : nd t = 110 min	(68)
**2**	Siemens Explora GN	1°) 110°C, 15 min 2°) 2 M HCl, 110°C, 15 min	Semi preparative RP-HPLC^e^ (H_2_O/CH_3_CN/TFA 80:20:0.1 8 mL/min)	RCY : 60% RCP > 98% A_m_ : 114 ± 60 GBq/µmol t = 90 min	(69)
**3**	FDG synthesizer	1°) 115°C, 15 min 2°) 3 M NaOH, 80°C, 5 min	SPE^f^	RCY 60 ± 5% RCP: 98% A_m_: 65.8 ± 10 GBq/µmol t = 35 min	(70)
**4**	Microfluidic (Advion)	1°) 190°C, 50 µL/min 2°) 3 M NaOH/MeOH/CH_2_Cl_2_ 45°C, 20 min	SPE^g^	RCY : 60% RCP > 98% A_m_ : nd t = 50 min	(71)

a octyldecyl silane column (Phenomenex).

b Nucleosil 100-7 C18 column, 250 × 16 mm (Macherey-Nagel).

c µBondapak C18 column, 10 µm, 300 x 7.8 mm (Waters).

d XBridge^®^ Prep C18 column, 5µm, 10 x 250 mm (Waters).

e Prodigy ODS-prep column, 10 µm, 10 x 250 mm (Phenomenex).

f C18 cartridge or Al_2_O_3_ cartridge and SCX cartridge.

g C18 Sep-Pak cartridge.

Several attempts were then performed to optimize this automated radiosynthesis process by varying the nature of the leaving group (tosylate *vs.* mesylate group), protecting groups (ethyl *vs. tert*-butyl esters) or purification method (RP-HPLC followed by SPE or SPE only). During these experiments, radiofluorination conditions remained similar (typically heating at 90-120°C in acetonitrile) and started from the same amount of precursor (4-5 mg).

After the radiofluorination step on precursor **1** at 90°C or 120°C for 15-20 min, hydrolysis was performed under acidic conditions using 2M or 3M HCl instead of TFA (66–68). Contrary to the previous method, heating at 115-120°C was necessary to achieve a full deprotection of the radiofluorinated intermediate. After purification by semi-preparative RP-HPLC and formulation by SPE (Sep-pak C18 or Oasis Plus HLB cartridge), d.c. RCY (26-39.8%) and RCP (> 99%) were finally similar to Reshef’s method. Despite comparable operating conditions, A_m_ were not homogeneous (25.3 – 235 GBq/µmol). This point could be due to the absence of UV-active chromophore of the chemical scaffold of the ML-10 compound leading to its challenging UV detection *via* analytical HPLC.

A second method started by a nucleophilic substitution at 110°C for 15 min of the tosylate precursor **2** (69). After cleavage of *tert*-butyl esters using 2 M HCl at 110°C for 15 min, [^18^F]ML-10 was obtained in good d.c. RCY (60%) and RCP greater than 98% after semi-preparative RP-HPLC purification and SPE formulation (Sep-pak C18 cartridge).

Similar results (d.c. RCY: 66%, RCP: 98%) were obtained starting from mesylate precursor **3** with a radiofluorination step performed at 115°C for 15 min followed by saponification of ethyl esters using 3 M NaOH at 80°C for 15 min (70). In this case, no RP-HPLC purification was performed and [^18^F]ML-10 was directly formulated *via* SPE. The authors demonstrated the SPE purification was more efficient when using a C18 cartridge instead of an alumina one (RCY: 60% *vs* 50% respectively). However, the use of ethanol was advantageously avoided for the elution of [^18^F]ML-10 from the alumina cartridge. Interestingly, this radiosynthesis was performed with a commercially available module used for [^18^F]FDG production, which is generally widely spread in nuclear medicine departments, and could facilitate the clinical production of [^18^F]ML-10.

Finally, Dewkar et al. developed an alternative radiosynthesis of [^18^F]ML-10 using a microfluidic system from tosylate precursor **4**. Such methodology usually requires very high reaction temperature (190°C) for the radiofluorination step, but allowed to produce the radiotracer with high d.c. RCY (60%) after a convenient SPE (Sep pak C18 cartridge) purification (71). Nevertheless, this faster methodology (50 min overall radiosynthesis duration) is still not used for daily radiopharmaceutical production of [^18^F]ML-10 in clinic.

In most cases, high d.c. RCY (60%) were obtained when the purification of [^18^F]ML-10 was performed by SPE only contrary to RCY obtained after HPLC purifications which were generally lower (23-60%). Moreover, overall radiosynthesis duration was shorter when no HPLC purification step was required (35-50 min *vs* 75-110 min respectively). It should be noted that, even if this HPLC purification step is time consuming, this method can lead to higher chemical purity and molar activity of the final radiotracer, which is an important criteria for radiopharmaceutical quality.

## Preclinical Studies

### Specificity of [^18^F]ML-10

The ability of ApoSense molecules, particularly ML-10, to detect apoptotic cells has been demonstrated in a series of *in vitro* experiments. The increased uptake of tritiated ML-10 was evaluated using cultured Jurkat cells (human T-cell leukemia lineage) treated with an anti-Fas antibody (63). A 9.2-fold increase in [^3^H]ML-10 uptake was detected compared to untreated cells. In addition, the radiotracer uptake was completely blocked by zVAD, a pan-caspase inhibitor, blocking the apoptotic process and demonstrating the direct relationship between ML-10 cell uptake and apoptosis.

For the radiofluorinated tracer, a 53% increase in radioactivity accumulation was demonstrated for cells treated with the anti-Fas antibody compared to untreated cells (72). In addition, it is interesting to note the selectivity of [^18^F]ML-10 for apoptotic cells compared to the radiotracer developed from the annexin V family, [^68^Ga]-Cys2-AnxA5, which shows a two-fold higher uptake into treated necrotic cells.

Finally, after *in vitro* validation of apoptosis induced by paclitaxel and epirubicin, a strong correlation between [^18^F]ML-10 cell uptake and the increase in the apoptotic fraction on two TNBC cell lines (MDA-MB-468 and MDA-MB-231) was evidenced, demonstrating the ability of this radiotracer to target cells in early apoptosis.

### Potential for *In Vivo* Imaging of Apoptotic Cells

Several preclinical studies were conducted in mouse and rat models to evaluate the ability of [^18^F]ML-10 PET imaging to detect apoptotic cells (**Table 3**).

**Table 3:** Preclinical studies of the use of [^18^F]ML-10 as apoptosis radiotracer or as tool for monitoring the therapeutic response.

Tumor type	Therapy	Delay before imaging	Images analysis	[^18^F]ML-10 uptake, correlation with therapy	Reference
Experimental cerebral stroke	Occlusion of middle cerebral artery	24h after induction of cerebral ischemia	% AI/g	Correlation with apoptotic fraction in ischemic territory	(65, 73)
Pulmonary fibrosis	Bleomycin	21 and 28 d after treatment	SUVmean	Uptake of [^18^F]ML-10 was proportional to the apoptotic fraction	(74)
Nasopharyngeal carcinoma	Radiotherapy or combination of radiotherapy and cetuximab	Before, 24 and 48 h after treatment	T/M	Uptake of [^18^F]ML-10 was proportional to the apoptotic fraction Increased T/M ratio after 24 and 48 h of irradiation or combination	(75, 76)
Head and neck squamous cell carcinomas	Doxorubicin	Before, 1, 3 and 7 d after treatment	T/L, %AI/g	Increased T/L ratio after 3 and 7 d of treatment	(77)
Non-Hodgkin’s lymphoma	Cyclophosphamide	Before and 24 h after treatment	SUVmean, SUVmax	No increased uptake of [^18^F]ML-10 after treatment	(72)
Triple negative breast cancer	Paclitaxel	Before, 1, 3 and 6 d after treatment	SUVmean, SUVmx, T/M	No increased uptake of [^18^F]ML-10 after treatment	(68)

In a first study, [^18^F]ML-10 PET imaging was performed 24 hours after occlusion of the middle cerebral artery in a mouse model of experimental cerebral stroke (73). The images highlighted an increased radiotracer uptake in areas of ischemia correlating with loci of DNA fragmentation, an important characteristic of apoptotic cells (TUNEL). Biodistribution studies also evidenced a fast radiotracer clearance from the blood, absence of radioactivity uptake in normal tissues and rapid renal elimination (65).

A second study, conducted in a rat model of pulmonary fibrosis, aimed at evaluating the potential of [^18^F]ML-10 PET imaging to follow the progression of pulmonary fibrosis after injection of bleomycin (5mg/kg). The authors showed that the accumulation of [^18^F]ML-10 in lung tissue increased proportionally with the level of apoptotic cells measured by flow cytometry (Annexin V-FITC; r² = 0.9863; p < 0.0001) and fibrotic activity (r² = 0.9631; p < 0.0001) (74).

### Evaluation of [^18^F]ML-10 PET Imaging for the Evaluation of Early Response During Chemotherapy in Murine Models

Several preclinical studies have evaluated [^18^F]ML-10 PET imaging as a biomarker of early response to cancer therapy (**Table 3**).

Different studies of human nasopharyngeal carcinoma xenografts models (CNE-1 and CNE-2) have evaluated the potential of [^18^F]ML-10 to predict xenograft radiosensitivity (75). After irradiation (15 Gy/mouse), CNE-2 xenografts showed a significant decrease in tumor volume four days after irradiation compared to the control group, which was not the case for the second model (CNE-1). PET imaging with [^18^F]ML-10 revealed an increase in the tumor uptake of radioactivity with a difference in tumor/muscle ratios (T/M) for the CNE-2 model after irradiation compared with the baseline. In contrast, the radiotracer uptake in the CNE-1 model was not altered.

Evaluation of the radiotracer was then carried out following treatment combining radiotherapy (15 Gy/mouse) and cetuximab administration (1 mg/mouse) (76). PET/CT scans showed a marked increase in the [^18^F]ML-10 tumor uptake 24 and 48 hours after these combined therapies. T/M ratio and SUVmax were significantly higher than those of the other three groups and the T/M ratio of [^18^F]ML-10 imaging showed a positive correlation of 0.926 with the apoptotic fraction (p < 0.001). The results of these two studies are encouraging as they demonstrate, in nasopharyngeal carcinomas, a high sensitivity and specificity of the radiotracer to predict early therapeutic response.

Demirci et al. evaluated the potential of [^18^F]ML-10 PET imaging in a subcutaneous UM-SCC-22B xenograft model receiving chemotherapy treatment (77). Mice received two doses of doxorubicin (10 mg/kg) with a 48 hours interval. PET imaging with [^18^F]ML-10 was performed 1, 3, and 7 days after treatment (5 control and 13 treated mice). PET imaging results showed a heterogeneous distribution of the radiotracer in the tumor. Tumor/liver ratio (T/L) analysis revealed an increase in the radiotracer uptake for the treated group compared to pre-treatment imaging on days 3 and 7 post-treatment (p < 0.05), but not on the day after treatment. T/L ratio in control mice did not evidence significant radiotracer uptake compared to baseline (p > 0.05). TUNEL analysis of tumor slices revealed a higher percentage of apoptotic cells in tumors from mice that received one or two treatments compared to control mice (p < 0.01). This study showed the potential of [^18^F]ML-10 PET imaging for early assessment of response. Nevertheless, further investigations seem mandatory, specially to ameliorate the acquisition and analysis of the images.

Another team evaluated [^18^F]ML-10 PET imaging, in comparison with two other radiotracers targeting apoptotic cells: [^68^Ga]-Cys2-AnxA5, and the iodinated analogue, [^123^I]ML-10, in a mouse lymphoma model (72). PET/MRI imaging using [^18^F]ML-10 was performed 24 hours after cyclophosphamide treatment (125 mg/kg). It is important to note that the tumor accumulation of [^18^F]ML-10 was low, so the volumes of interest were segmented on magnetic resonance imaging (MRI) and transferred to PET imaging. No significant increase in SUVmean and SUVmax was highlighted following treatment compared to pre-treatment imaging. However, autoradiography of tumor sections showed a specific uptake of [^18^F]ML-10 in the regions containing apoptotic cells following treatment. TUNEL analysis of the tumor slices evidenced a two-fold increase in the number of apoptotic cells measured 24 hours after treatment. The authors concluded that [^18^F]ML-10 was able to detect apoptotic cells at the tumor level in their model. However, [^18^F]ML-10 PET imaging before and after treatment failed to detect an apoptotic response following cyclophosphamide treatment. The authors explained this failure by the pH of the tumor microenvironment, which is inherently more acidic, and the insufficient number of apoptotic cells to cause a significant increase in [^18^F]ML-10 uptake.

Finally, we evaluated the tumor uptake of [^18^F]ML-10 in a mouse model of triple negative breast cancer (MDA-MB-468 and MDA-MB-231) (68). The main objective of this study was to confirm the ability of the [^18^F]ML-10 radiotracer to demonstrate the increase in the apoptotic fraction following treatment with paclitaxel. *In vivo* PET imaging of “untreated” xenografts then showed a significant correlation between tumor apoptotic fraction and radiotracer uptake in this tumor model harboring basal apoptotic fraction (non-treatment-induced). In addition, no binding of the radiotracer was reported on healthy tissues. Nevertheless, tumor apoptosis induced by paclitaxel treatment did not result in a significant increase in the [^18^F]ML-10 tumor uptake after one or two doses of paclitaxel (20 mg/kg), while [^18^F]FDG uptake, reflecting tumor metabolism, decreased over time. Several hypotheses were then formulated, the main one being the sensitivity of the radiotracer uptake to pH variations, which may be significant at the tumor level during chemotherapy treatment. The demonstration of a correlation between hypoxia and [^18^F]ML-10 accumulation and the influence of extracellular pH on *in vitro* radiotracer uptake supported this hypothesis. Evaluations were conducted on apoptotic MDA-MB-468 cells (TNBC) after 72 hours of treatment (10-fold IC_50_ dose of paclitaxel) and incubation with [^18^F]ML-10. Acidification of the pH of the culture medium (pH 5.9 - 8.0) increased the uptake of [^18^F]ML-10. Therefore, the predictive value of [^18^F]ML-10, could be influenced by the level of tumor hypoxia in the model and by the capacity of the different chemotherapies to impact the extracellular pH.

### Voxel-Based Analysis

A new approach called quantitative voxel-by-voxel method and initially developed by Moffat and Ross, for MRI analysis, has been used for [^18^F]ML-10 PET imaging analyses. This algorithm considers the observed spatial heterogeneity in tumor response and may be an early indicator of response to tumor treatment (78). Thus, for the voxel-by-voxel parametric of [^18^F]ML-10 PET analysis, the change in the apoptotic signal (i.e. the fixation of each pixel belonging to a region of interest) within a tumor after treatment can be compared to the signal before treatment. To ensure that each voxel corresponds to the same anatomical region, an optimal resetting of the images, called spatial normalization, is mandatory. This subtraction of the signal intensity for each voxel allows the production of a statistical parametric map representing the changes that occurred voxel by voxel as a result of the treatment. The average percentage change of all voxels is then plotted to classify the voxels into three categories:

- voxels with a positive signal, defined as an increase in the radiotracer uptake of more than 12.5% from baseline. Biologically, these values represent cells in the early stages of apoptosis, thus showing an increased uptake of [^18^F]ML-10.

- voxels with a negative value, defined as a decrease in radioactivity uptake greater than 12.5%.

- voxels showing no change, defined as a change in the signal not exceeding 12.5%. The concept of a threshold value, distinguishing between these three zones was adapted from the studies of Moffat et al. (78).

The uptake of [^18^F]ML-10 by the tumor thus leads to a characteristic variance in the voxel-to-voxel signal, corresponding to a specific “signature” of each tumor. This “signature” is expected to change when an effective treatment is administered to the patient, whereas no significant change should be noticed if case of treatment failure. The measurement of the variance of tumor uptake of [^18^F]ML-10 induced by treatment, based on variations within voxels, may allow early discrimination between sensitive and non-sensitive tumors, and thus be predictive of subsequent changes (decrease in tumor volume), normally observed only after several weeks.

## Clinical Studies Using Pet Imaging

Initially, ApoSense’s plan was to develop [^18^F]ML-10 as a diagnostic PET radiotracer for the detection of active lesions in relapsing-remitting multiple sclerosis, *via* the targeting their characteristic apoptotic cells. Then, ApoSense shifted its research interest on the study of [^18^F]ML-10 in oncology as a potential radiotracer of apoptosis for the early detection of patients treated with radiations or chemotherapy. Clinical trials using [^18^F]ML-10 are listed in **Table 4**. Many of them were initiated in cancers with unmet clinical need, as glioblastoma or brain metastases, for which conventional imaging failed to detect recurrence or asses therapeutic response with high accuracy.

**Table 4:** Clinical studies of the use of [^18^F]ML-10 for apoptosis tracer or monitoring therapeutic response.

Indications	Clinical study	Therapy	Delay before imaging	Images analysis	[^18^F]ML-10 uptake, correlation with therapy	Reference
Healthy humans	Phase I	none	–	SUVmean	Uptake of [^18^F]ML-10 in apoptotic cells in testicular tissue	(79)
Acute ischemic cerebral stroke	Phase II	none	2 at 6 d after acute ischemic cerebral stroke	-	Uptake of [^18^F]ML-10 in ischemic territory	(80)
Brain metastases	Phase IIa	Radiotherapy	Before and after 9 or 10 fractions of radiotherapy	Voxel-based analysis	Correlation with tumor response	(81) NCT00791063
Brain metastases	Phase IIb Aposense Ltd.	Radiosurgery	Before and 48 h after therapy	Voxel-based analysis	Correlation with tumor response	(82) NCT00696943

### Physiological Distribution of [^18^F]ML-10 in Humans

In a Phase I trial on healthy volunteers, [^18^F]ML-10 showed high *in vivo* stability with favorable biodistribution and dosimetry profiles (EudraCT 2006-001288-4) (79). This non-randomized open study included eight subjects (four men and four women) who received a single dose of [^18^F]ML-10. Administration was followed by a PET/CT imaging sequence up to 220 minutes post-injection to assess the *in vivo* stability of the radiotracer (i.e. search for metabolites) and a dosimetry study using OLINDA/EXM 1.0 software to estimate the radiation doses absorbed by the various organs. [^18^F]ML-10 was very stable *in vivo* with more than 97.5% of the unchanged radiotracer detected in plasma after 150 minutes post injection. Evaluation of the biodistribution of the radiotracer showed a rapid distribution of [^18^F]ML-10 in the extracellular space, an absence of uptake in non-target organs, and a rapid urinary clearance.

The dosimetry study indicated that the doses delivered to the organs were comparable to other well-known fluorinated PET radiotracers. No tracer-related adverse effects were observed throughout the study. In addition, the behavior of the radiotracer for the detection of apoptotic cells was observed in the four men enrolled in the clinical trial. Indeed, apoptosis is known to play an important role in testicular physiology, ensuring the elimination of defective germ cells. Histological studies carried out on sections of young adult male testes showed that 5-10% of the cells were apoptotic (83). The accumulation profile of [^18^F]ML-10 in the testes highlighted an increase in the radiotracer uptake following injection, peaking around 25 min (SUV max = 2), then a plateau up to 90 min before a slow uptake decrease. The signal intensity in the testis was five-fold higher than in the muscle.

### Acute Ischemic Cerebral Stroke

In an open-label Phase II study, [^18^F]ML-10 was evaluated as a radiotracer for PET imaging of apoptosis in patients with acute ischemic stroke (80). A total of eight patients received a single intravenous dose of [^18^F]ML-10 administered 2.5 to 6 days after the onset of stroke. Three consecutive PET/CT scans were performed within 220 minutes of radiotracer administration, each lasting 30-45 minutes. All these images showed a selective uptake of [^18^F]ML-10 in the regions of the infarction. Follow-up of these patients did not reveal any side effects caused by the radiotracer injection.

### Brain Metastases

In a Phase IIa clinical study (NCT00791063) in subjects with brain metastases treated with radiotherapy, [^18^F]ML-10 uptake was evaluated by PET/CT imaging in 8/10 patients (81). The initial histological diagnosis of these metastatic patients was non-small cell lung cancer, small cell lung cancer, uterine sarcoma, melanoma, and breast cancer. These patients received a dose of 30 Gy in 10 fractions over 2 weeks. PET/CT imaging was performed before the radiotherapy and 9 to 10 days after the start of the treatment. Analysis of the images revealed heterogeneity in the [^18^F]ML-10 uptake, corresponding to the diversity of cellular responses. Voxel-based analysis of the PET images showed a radiotracer uptake change of approximately 69.9% (36.3% - 100%), highlighting a significant correlation (Pearson’s analysis r = 0.9) between the change in radiotracer uptake and the decrease in the tumor volume observed two months after treatment.

The objective of a second phase II trial was to evaluate the potential of [^18^F]ML-10 for imaging apoptosis in 29 patients with brain metastases treated with stereotactic radiosurgery (82). Analysis of the raw images, registered 48 hours post-treatment, revealed a significant increase in the uptake of [^18^F]ML-10. To complete this analysis, the authors used a subtraction analysis of “post-radiotherapy”-“pre-radiotherapy” images and revealed a larger central apoptotic zone compared to the periphery of the tumor for these patients.

However, subtraction analysis did not evidence the heterogeneity and overall changes in radiotracer uptake. A quantitative analysis based on voxel-by-voxel changes was used to further investigate apoptotic changes within the tumor. The signal variation in each voxel within the volume of interest was plotted on a scatter plot and a higher number of “apoptotic” voxels in responder patients was evidenced. In addition, the analysis revealed a strong correlation between the change in the [^18^F]ML-10 uptake and the change in the estimated tumor volume 2 to 4 months after the end of treatment (Pearson r² = 0.862; p < 0.05). The authors concluded that voxel-based analysis of [^18^F]ML-10 PET/CT images allowed early assessment of the therapeutic response after radiation therapy.

### Glioblastoma

In a case study published by the Oborski’s team, a glioblastoma patient treated with radiation therapy combined with temozolomide (75 mg/m²) underwent two [^18^F]ML-10 PET scans before and 3 weeks after the start of the treatment (84). Analysis of the images showed an increase in the radiotracer uptake compared to the start of treatment. Histological analyses of the tumor tissue confirmed the presence of apoptotic cells correlating with the location of the [^18^F]ML-10 uptake.

Subsequently, Oborski’s team evaluated a novel approach to study the response to therapy with [^18^F]ML-10 PET imaging of apoptosis supported by MRI follow-up in four patients with glioblastoma. Images were acquired before and 2 to 3 weeks after baseline (85, 86). The results of these two studies did not show the expected results. [^18^F]ML-10 uptake could not clearly be related to the disease progression. The authors believe that the aggressiveness of glioblastoma was responsible for this failure because proliferation and cell death were simultaneous.

## Discussion

For several years, molecular imaging has been increasingly used in the field of oncology, particularly [^18^F]FDG PET imaging to evaluate glucose metabolism mainly during the diagnostic phase but also for the follow-up of treatments in many cancers, in particular lymphoma. However, the sensitivity and specificity of [^18^F]FDG remains insufficient and complementary imaging techniques, such as apoptosis imaging, would be of value. This would allow the discrimination of apoptotic cells (24 - 48 hours post-treatment) from metabolically inactive cells (a few weeks post-treatment) to reduce the number of “false positive” [^18^F]FDG PET findings. In such context, the development of a novel PET radiotracer specific of apoptosis will be of great interest.

In recent years, several new radiotracers have been developed as apoptosis imaging agents. One of them, [^18^F]ML-10, presented interesting results especially regarding its specificity in distinguishing apoptotic from necrotic cells and its very rapid clearance from non-target organs. The advantage of [^18^F]ML-10, compared to other radiotracers targeting apoptosis (radiolabeled Annexin V and radiolabeled Caspase-3), is a rapid biodistribution due to its low molecular weight and hydrophilicity which allows early tumor imaging including brain cancer. In this review, we examined the current state of research data available on [^18^F]ML-10. Several research groups have investigated the feasibility of using this radiotracer through preclinical and clinical studies.

### Early Assessment of the Response to Chemotherapy Using [^18^F]ML-10

Three different aspects of the [^18^F]ML-10 PET imaging were reported the literature. Firstly, the ability of the [^18^F]ML-10 to visualize apoptotic cells. Initial preclinical and clinical results highlighted a good correlation between radiotracer uptake and the proportion of apoptotic cells. Moreover, this radiotracer can specifically accumulate in apoptotic cells compared to necrotic cells (72). However, some research groups have reported difficulties in interpreting the uptake of [^18^F]ML-10 in tumors, showing a weak accumulation which can highly complicate the interpretation of obtained images. To circumvent this problem, a voxel-based analysis has been developed during clinical trials (81) and allowed to visualize the variation of the voxel by voxel signal between pre-treatment and post-treatment imaging and to build a classification in three categories: increase, decrease or stability of the signal. Responder patients had a statistically higher number of voxels with increased radiotracer accumulation. The voxel-based analysis was developed to discriminate some images of patients to address the spatial heterogeneity observed in tumor responsiveness. This method of analysis has never been used in preclinical studies because it requires a high quality image registration and resolution of the acquisition devices. One of the negative points of all the studies carried out is the non-uniformity of the image acquisition parameters (post-injection imaging time, etc.) and their interpretation (SUVmean, T/M, %AI/g, voxel-based analysis, etc). Finally, another interesting point to be discussed concerns the radiotracer ability to predict the complete histological early response to therapy. Indeed, the results found in the literature are controversial. Clinical and preclinical studies concerning radiotherapy treatments alone or in combination with chemotherapy have revealed promising results with a strong potential of the radiotracer for PET imaging of apoptosis and its use in clinical routine. However, the results of two other preclinical studies showed no correlation between tracer uptake and the apoptotic fraction measured in the tumor in a mouse lymphoma model treated with cyclophosphamide and in a TNBC model treated with paclitaxel (68, 72). Several hypotheses were raised by the authors. The first one is that [^18^F]ML-10 is unable to detect a low increase in the number of apoptotic cells. In view of the various in vitro studies concerning cell uptake assays, this hypothesis is unlikely. The second hypothesis is related to the variation in the extracellular pH during chemotherapy which can highly influence the tumor uptake of [^18^F]ML-10 (80, 81, 87, 88). At physiological pH, the di-anionic form of malonate predominates (pKa values of 5.78 and 3.07). A decrease in extracellular pH favors the formation of the mono-protonated carboxylic acid form and facilitates its interaction with the membrane of apoptotic cells [183]. Hypoxia and apoptosis in solid tumors can lead to acidification of the extracellular medium. In addition, some studies have demonstrated the ability of cancer cells to reverse the pH gradient across the membrane cell, which induces a decrease in extracellular pH and facilitates the uptake of [^18^F]ML-10 (88–91). This hypothesis is supported by the correlation between the fixation of [^18^F]ML-10 and [^18^F]FMISO observed in the TNBC model (68). In vitro experiments studying the impact of extracellular pH variation on radiotracer uptake, reported in our paper, support this mechanism. In addition, the study showed that chemotherapy treatment leads to an increase in tumor pH (92), that could explain the decrease in cell uptake of [^18^F]ML-10 in this model. However, this phenomenon was not observed in the orthotopic model. Zhang’s team was able to show that orthotopic tumor implantation resulted in a more favorable and homogeneous tumor growth and an increase in the density of the microvessels (87). Probably, better tumor vascularization then allows better elimination of necrotic cells and therefore less variation of the pH. However, even in this model, no significant increase in tumor uptake of [^18^F]ML-10 was observed during treatment with paclitaxel. These results showed that [^18^F]ML-10 is not enough sensitive to assess the therapeutic response to chemotherapy in vivo, probably due to the change in extracellular pH values. However, the ability of [^18^F]ML-10 to predict the therapeutic response has been confirmed in several studies when used in combination with radiotherapy (82, 84). Radiotherapy treatment is known to induce, in addition to the “cell death” effect, inflammation in the tumor, which changes extracellular pH via: (i) activation of the immune system, resulting in an increased blood flow, and (ii) acidification of the microenvironment due to the presence of immune cells and cytokines (93). The tumor uptake of [^18^F]ML-10 during radiotherapy treatment is thus favored in such microenvironment. In summary, the **Table 5** lists the few clinical indications for performing [^18^F]ML-10 PET imaging and the imaging conditions.

**Table 5:** Clinical use of [^18^F]ML-10 PET imaging.

Applications	[^18^F]ML-10 PET imaging	Requirements
Assessment of acute ischemic cerebral stroke.	Yes	2,5 to 6 days after onset.
Assessment of the early response to radiotherapy in brain metastasis.	Yes	48 hours post-treatment. Voxel-based analysis.
Assessment of the early response to chemotherapy in solid tumor.	No	

### Apoptosis, an Interesting Biological Target?

Numerous radiotracers targeting apoptosis have been developed, but none of them are used in clinical practice. The apoptosis phenomenon is a rapid biological mechanism, influenced by many parameters that may explain the disappointment of current radiotracers. During therapy, several changes occur at the vascular and cellular level within the tumor:

- an alteration in the permeability of the vessels that can influence the intratumoral biodistribution of the radiotracer,

- a change in the microenvironment influencing the pH,

- the appearance of inflammatory areas that may alter the radiotracer’s ability to target apoptotic cells.

In addition, tumor phenotypic heterogeneity favors variations in tumor response to various stimuli. This heterogeneity can be found at different levels: between cancers of the same subtype, between the primary tumor and related metastases. Finally, depending on the therapeutic regimen used, the time to apoptosis onset and the proportion of apoptosis among other types of cell death are variable. All these parameters must be considered to define the optimal time for imaging of apoptosis. Due to these considerations, the relevance of apoptosis as a target for the early evaluation of the therapeutic response can be called into question (94).

Concerning the imaging of “dead cells”, the question to ask is: should only apoptosis be targeted, with all the constraints previously stated, or should we find a cell death radiotracer capable of targeting all types of cell deaths? Taking into consideration the different mechanisms of cell death, it does not seem possible to develop such a tracer able to challenge all death process. Therefore, additional studies are necessary in order to identify, in a first step, a sensitive and specific radiotracer of apoptosis. Then, in a second step, it is necessary to determine a threshold of the number of apoptotic cells from which it would be possible to predict the complete histological response in a neoadjuvant situation. Nevertheless, several questions remain whatever the parameter under study is. What is the detection threshold allowing the therapeutic regimen to be modified? Is the variation in cellular metabolism the right parameter? What is the best time to perform intermediate imaging? Is it necessary to carry out intermediate PET imaging with 1 or 2 cures?

### What Is the Best Analysis Method to Evaluate the Therapeutic Response With PET Imaging?

Many strategies, ranging from visual reading to complex approaches such as textural, radiomics or deep learning strategies have been investigated to evaluate the therapeutic response.

The visual analysis method comparing baseline and interim PET has proven its robustness and prognostic value for lymphoma. The Deauville 5PS scale was the first PET method to have such a high impact on the management of cancer (95). It was a long step by step process of validation with a final end-point to readapt therapy according to PET results. It is quite obvious that this success story is not transposable to solid tumors. Assessment of the therapeutic response using PET imaging still remains in research phase. Guidelines advising visual and SUV analysis have been published since 1999, however they are still not recommended for clinical practice (5, 96). Even if concepts have recently evolved, solid tumors are more heterogeneous than hematology disease (97). This cellular heterogeneity could contribute to explain the difficulty to differentiate responders from non-responders when conducting a visual or quantitative standard method. For instance, a voxel-based parametric approach had to be performed in order to accurately separate the responders in a population of cancers treated with chemotherapy and assessed with [^18^F]ML-10 apoptotic tracer (79). Before and after therapy, tumor is made of necrotic, apoptotic and living (i.e. resistant) cells. A standard parameter such as SUV could not accurately sort out cells going from one state to another. Considering clonal heterogeneity, a radiomic approach with textural features of images or a deep learning algorithm seems to be more efficient (98). Artificial intelligence will possibly offer the opportunity to stratify patients whatever the tracer and type of treatment used, including emergent therapies.

### Alternative Strategies to Overcome the Limited Applicability of [^18^F]ML-10 PET Imaging Is Not the Right Candidate for Early Assessment of Therapeutic Response in Solid Tumors

For the imaging of living cells, several strategies can be used. Among them, the first and most studied one is the imaging of glucose metabolism using [^18^F]FDG. As mentioned above, there are some limitations regarding the lack of consensus on the optimal time for intermediate imaging as well as the metabolic criteria to use in order to define the tumor response. In addition, the metabolic response is strongly influenced by the phenotype of the tumor cells. Thus, the evaluation criteria must be adapted to each clinical situation (phenotype and treatment). Other tracers for the imaging of living cells have been developed such as [^18^F]fluorocholine used clinically in prostatic adenocarcinoma (French Marketing Authorisation). This radiopharmaceutical metabolism is identical to the one of choline. Compared to [^18^F]FDG, [^18^F]fluorocholine advantageously accumulates in cells with low proliferation rates. Nevertheless, this radiotracer presents a strong hepatic and digestive elimination making the interpretation of some images difficult and can induce false positives in inflammatory areas. Moreover, in the case of some cancer such as TNBC, the use of [^18^F]fluorocholine is limited as these highly proliferative tumors efficiently capture [^18^F]FDG. Another metabolism tracer is [^18^F]fluciclovine, a leucine analogue, which is transported across mammalian cell membranes by amino acid transporters such as LAT-1 and ASCT2. This molecule has recently obtained the French Marketing Authorisation for the evaluation of recurrence in prostate cancer. A pilot study on invasive ductal and invasive lobular breast cancer patients has shown a strong correlation between variations in the radiotracer accumulation and therapeutic response (99). Finally, imaging of living cells can be carried out using a radiotracer of cell proliferation such as the 3’-deoxy-3’-[^18^F]fluorothymidine ([^18^F]FLT). Its main advantage over [^18^F]FDG is the absence of binding to inflammatory areas, which are often significant at the start of the treatment (100). A recent clinical study has shown the potential of [^18^F]FLT PET imaging as an early marker of response to the neoadjuvant therapy (101). Considering this, it would be interesting to conduct studies to assess the potential of [^18^F]FLT and [^18^F]-fluciclovine compared to [^18^F]FDG in solid tumors. Phase II and III studies will be necessary to confirm the interest of early therapeutic assessment by molecular imaging. An innovative approach to evaluate early response would be to study the mechanisms of early tumor chemoresistance. The identification of predictive and explanatory molecular signatures of tumor chemoresistance would allow the development of predictive tracers of tumor chemoresistance. Therefore, the identification of the biomarker(s) representative of this resistance is still challenging.

Finally, a new radiotracer named [^68^Ga]-labeled fibroblast activation protein inhibitor (FAPI) has made a breakthrough in nuclear medicine. This promising PET radiotracer has shown great potential in many types of cancers (102, 103). Whether this tracer would be able to detect early and accurately the response to conventional chemotherapy has to be explored.

## Conclusion

In conclusion, several research groups have investigated different aspects of [^18^F]ML-10, and the published reports highlighted conflicting results summarized in this review article, particularly the inability of the radiotracer to image *in vivo* apoptosis induced by chemotherapy treatments. Hence, future developments should be focused on the identification of more specific PET imaging radiotracers for the early assessment of therapeutic response in oncology.

## Author Contributions

This review was drafted by EJ and SS, and critically revised by EC, AM-B, FP-L, and FC. All authors contributed to the article and approved the submitted version.

## Conflict of Interest

The authors declare that the research was conducted in the absence of any commercial or financial relationships that could be construed as a potential conflict of interest.

## Publisher’s Note

All claims expressed in this article are solely those of the authors and do not necessarily represent those of their affiliated organizations, or those of the publisher, the editors and the reviewers. Any product that may be evaluated in this article, or claim that may be made by its manufacturer, is not guaranteed or endorsed by the publisher.
